# Lipoid Pneumonia in a Gas Station Attendant

**DOI:** 10.1155/2014/358761

**Published:** 2014-10-08

**Authors:** Gladis Isabel Yampara Guarachi, Valeria Barbosa Moreira, Angela Santos Ferreira, Selma M. De A. Sias, Cristovão C. Rodrigues, Graça Helena M. do C. Teixeira

**Affiliations:** Department of Pulmonology, Faculty of Medicine, Fluminense Federal University, Pedro Antonio University Hospital, Rua Marques de Paraná, 303 Center, 24033-900 Niterói, RJ, Brazil

## Abstract

The exogenous lipoid pneumonia, uncommon in adults, is the result of the inhalation and/or aspiration of lipid material into the tracheobronchial tree. This is often confused with bacterial pneumonia and pulmonary tuberculosis due to a nonspecific clinical and radiologic picture. It presents acutely or chronically and may result in pulmonary fibrosis. We describe here a case of lipoid pneumonia in a gas station attendant who siphoned gasoline to fill motorcycles; he was hospitalized due to presenting with a respiratory infection that was hard to resolve. The patient underwent bronchoscopy with bronchoalveolar lavage, which, on cytochemical (oil red O) evaluation, was slightly positive for lipid material in the foamy cytoplasm of alveolar macrophages. Due to his occupational history and radiographic abnormalities suggestive of lipoid pneumonia, a lung biopsy was performed to confirm the diagnosis. The patient was serially treated with segmental lung lavage and showed clinical, functional, and radiological improvement.

## 1. Introduction

The occurrence of exogenous lipoid pneumonia (LP) in healthy adults is infrequent, occurring mainly in occupational accidents, resulting in microaspiration of lipid formulations [1, 2]. These oily substances are not cleared by the lung and inhibit the cough reflex and function of the mucociliary apparatus, which facilitates aspiration, even in normal individuals [3]. They are also responsible for recurrent acute respiratory infections. Diagnosis is often difficult because it mimics other common pulmonary diseases, such as bacterial pneumonia and pulmonary tuberculosis [1].

The objective of this work was to report on the clinical course and treatment of a case of exogenous LP in a gas station attendant who siphoned gasoline in filling motorcycles.

## 2. Case Report

**Figure 1:**
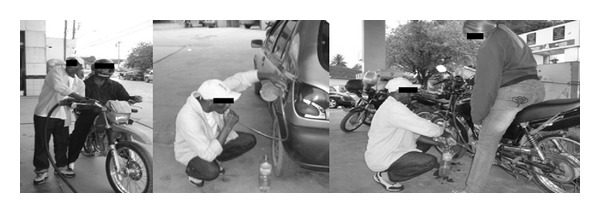
Patient siphoning excess gasoline in filling vehicles due to wrong information provided by the clients.

A 41-year-old man, gas station attendant for 14 years, reported that he frequently siphoned gasoline while filling vehicles, mainly motorcycles (Figure 1). One year ago start makes dry cough and nonspecific pain insidiously in the lower third of the left hemithorax, with progressive worsening of symptoms. He denied any history of fever or weight loss. He sought medical attention, where he was hospitalized for 15 days for treatment of community-acquired pneumonia. Since there was no improvement, empirical treatment for pulmonary tuberculosis was implemented, also without response, and he was therefore referred to the Respiratory Outpatient Clinic of Hospital Universitario Antonio Pedro (HUAP) with the same symptoms, besides dyspnea on exertion. He denied prior history of tobacco use and other pulmonary diseases.

**Figure 2:**
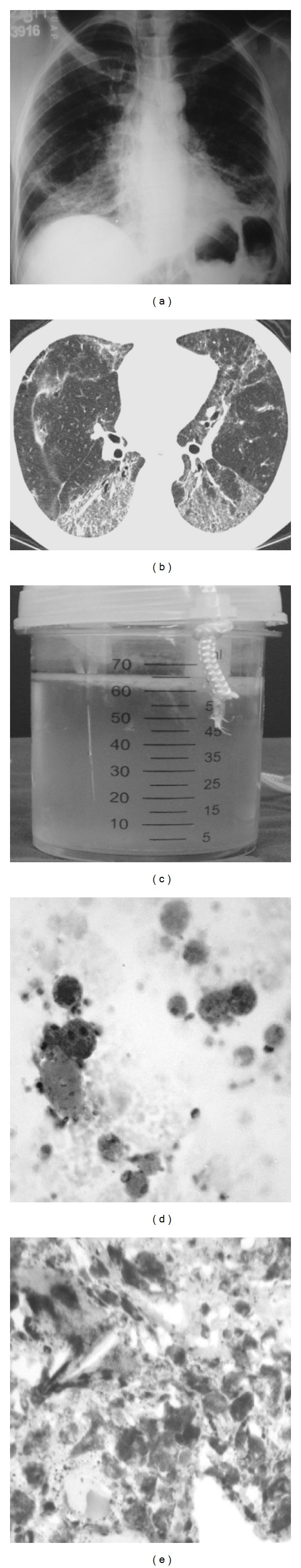
(a) PA chest radiograph: consolidations at lung bases. (b) Chest HRCT: nonhomogeneous consolidations, ground-glass opacities, areas of fibrosis with parenchymal beams, and bronchiectasis traction. (c) Segmental pulmonary lavage fluid: cloudy with halo of fatty supernatant. (d) Bronchoalveolar lavage fluid: presence of macrophages with foamy cytoplasm showing positive oil red O staining. (e) Histopathologic section of lung (oil red O, 400x) showing orange-colored lipid contents “lipid laden macrophages.”

Physical examination revealed good general condition, afebrile, with crackling rales at the lung bases and clubbing. Hemogram and blood chemistries were normal and the PPD was negative. A chest radiograph revealed consolidations in the lung bases. High resolution computed tomography (HRCT) of the chest showed, despite the extensive nonhomogeneous consolidations in the posterior segments of both lower lobes, ground-glass opacities and areas of fibrosis with bronchiectasis in the lung parenchyma (Figures 2(a) and 2(b)). Spirometry revealed moderate restrictive ventilatory disturbances and 6-minute walk distance was 420 m (maximum and minimum: 608 and 455 m). The patient was subjected to bronchofibroscopy with bronchoalveolar lavage, where cytology revealed pleocytosis with a predominant increase in the percentage of lymphocytes (57%). Microbiological (BK, fungi, and bacteria) and cytopathologic studies were negative. Cytochemical evaluation with oil red O showed weak positive staining, which called for a lung biopsy. Histopathologic assessment of the lung fragment revealed distortion of the pulmonary architecture with fibrosis and multinucleated giant cells with cholesterol clefts and intra-alveolar and interstitial macrophages showing foamy cytoplasm stained with oil red O “lipid laden macrophages,” confirming the lipid nature, compatible with exogenous LP (Figure 2(c)).

The patient underwent segmental pulmonary lavage (Table 1) series made with warm physiological saline 0.9% with a volume of 100 mL per segment in the areas of greater commitment demonstrated by tomography of chest high-resolution, three segments per procedure. The aim of the lung lavage segment was to improve respiratory symptoms and changes in cellularity of the liquid were made ten sessions, once a week, associated with the use of corticosteroids (prednisone 1 mg/kg/day orally) for one year to wean gradual, taking it to present significant clinical and radiological improvement (Figure 2(e)).

## 3. Discussion

LP was described for the first time by Laughlen in 1925, where, according to its origin, it can be endogenous, exogenous, or idiopathic [1, 3].

The rarer endogenous form is found associated with pulmonary fat embolism, alveolar proteinosis, and lipid deposit diseases such as alveolar phospholipoproteinosis, Niemann-Pick disease, Wegener granulomatosis, and undifferentiated connective tissue disease. The idiopathic form, also rare, has been described in healthy smokers [1–3].

The exogenous form is often more common in children and the elderly, and it is related to the use of mineral oil for the treatment of intestinal constipation.

Mineral oil, inert material for our body, reduces glare from coughing or choking, facilitating aspiration, even in the absence of risk factors. In the lung, mineral oil is phagocytized by the macrophages and fills the alveoli, remaining in the alveolar walls and reaching the interlobular septa through lymphatic channels, which results in foreign body-type granulomas localized in the pulmonary interstitium, and later, with repeated aspirations, this can develop into pulmonary fibrosis and loss of lung function and volume [1, 4].

Exogenous PL can present in the acute or chronic form. The acute form is described in children and the elderly in the treatment of intestinal constipation.

The chronic form is less frequent and occurs as a consequence of continuous aspiration of various materials in the work area (contact with oil vapors, kerosene, and/or others), as in the case described here, in which the patient, a gas station attendant, siphoned excess gasoline (petroleum derivate like mineral oil).

Other related exposures, also not very common, include the inhalation of nasal preparations for nasopharyngeal obstruction and chronic use of Vicks Vaporub [1–5].

The differentiation between the exogenous and endogenous forms is made not only by the clinical history compatible with the ingestion and/or aspiration of oils, in the case of the exogenous form, but also by the distinct histologic characteristics, that is, the detection of extracellular lipid material, the appearance of intracytoplasmic vacuoles, the distribution of macrophages in the lung tissue, and the physicochemical characteristics of the oil [1, 4]. The degree of damage and pulmonary fibrosis depends on the quantity of free fatty acid and the rapidity of the process of hydrolysis at the alveolar level. The different characteristics of the oils can be detected according to histochemical reactions: mineral oil in exogenous PL shows a positive reaction as a yellow or orange color by staining with Sudan IV and oil red O, while in endogenous PL, staining shows a positive reaction with a red color [4].

In exogenous PL, the clinical manifestations are nonspecific, varying according to the age of the patient and the form of exposure (acute or chronic). Usually, patients present with cough and dyspnea [4]. Fever, weight loss, chest pain, and hemoptysis are less frequent manifestations [1, 3, 4]. The majority of exogenous PL cases are initially treated as bacterial pneumonia and occasionally also as pulmonary tuberculosis due to clinical, laboratory, and nonspecific radiologic findings [1, 4, 6].

In the present case report, the patient was also initially treated for pneumonia and tuberculosis without clinical and radiologic improvement.

The radiologic alterations of exogenous PL are nonspecific, varying from perihilar opacity to extensive areas of consolidations with air bronchogram, which occur mainly in the lower and posterior lobes of the lungs, resulting in similar appearance as lobar pneumonia [6]. Accordingly, it is important to include exogenous PL in the differential diagnosis of the chronic, repeated, or delayed pneumonia.

HRCT is the best imaging method for the diagnosis of exogenous LP, where its main finding is alveolar consolidation with negative density (−30 to −150 Hounsfield units), frequently associated with the presence of fat, ground-glass opacities, abnormalities of the interstitium, and nodular lesions (small poorly defined centrilobular nodules) [4, 6].

The diagnosis is confirmed by clinical history of ingestion and/or aspiration of mineral oil and the presence of alveolar macrophages with foamy cytoplasm and positive cytochemical staining with Sudan or oil red O in the sputum, bronchoalveolar lavage fluid, and gastric lavage fluid or tissue. In the patient reported here, the diagnosis of exogenous LP was suspected because of his occupational history and confirmed by histopathologic examination of the lung fragment, which showed cytochemical staining with oil red O, indicating the presence of lipid material in the cytoplasm of the macrophages “lipid laden macrophages.”

There is general consensus that the principal approach in the treatment of exogenous LP is the immediate suspension of mineral oil in cases of children and the elderly. However, in adults, staying away from the work area and further exposure is urged, such as for the patient described here.

The use of corticosteroids is still controversial in the treatment of PL, where they are recommended in the more serious cases, as a strategy to block inflammation and the development of fibrosis [3, 4].

Later studies suggested that the principal measure would be the mechanical removal of the oil present in the lungs, in view that natural defense mechanisms, such as mucociliary activity and cough are harmed devido the presence of mineral oil, preventing its removal. There are reports of PL treated successfully utilizing total pulmonary lavage in patients who did not respond to treatment with high doses of corticosteroids. Sias et al. [4] utilized multiple segmental pulmonary lavages in 10 children with PL obtaining clinical and radiologic improvement. Multiple segmental pulmonary lavages have the advantage of not needing general anesthesia and can be done in cases in which total pulmonary lavage shows more risk than benefit for the patients.

The patient in the present case report was subjected to sequential segmental pulmonary lavage combined with systemic corticotherapy, resulting in clinical, functional, and radiologic improvement.

The scheme used steroids with prednisone 1 mg/kg/day, orally during gradual weaning year every three months (60 mg, 40 mg, 20 mg, and 10 mg), which showed good results.

## 4. Conclusion

Chronic exogenous PL in healthy adults is an unusual condition and its diagnosis can be delayed, since the clinical picture and radiologic changes can mimic bacterial pneumonia and tuberculosis. The occupational history is of extreme importance and should always be investigated. Avoidance of the exposure to mineral oils is the main treatment of exogenous LP. Other treatment options described in literature include whole lung lavage, lobar or segmental lavage, and corticosteroids for selected severe cases.

## Figures and Tables

**Table 1:** Global and specific cytology of bronchoalveolar lavage fluid, segmental and sequential (evolution), after 10 sessions.

	Total cells/mm^3^	Macrophages %	Lymphocytes %	Neutrophils %	Eosinophils %
Normal range	200 to 250	85 to 92	6 to 12	1 to 3	<1
Before	382	30	57	10	3
After	152	70	24	5	1
